# Dramatic regression and bleeding of a duodenal GIST during preoperative imatinib therapy: case report and review

**DOI:** 10.1186/1477-7819-8-47

**Published:** 2010-06-02

**Authors:** Andreas Hecker, Birgit Hecker, Birgit Bassaly, Markus Hirschburger, Thilo Schwandner, Hermann Janßen, Windried Padberg

**Affiliations:** 1 Department of General and Thoracic Surgery, University Hospital of Gießen, Rudolf-Buchheim-Street 7, 35392 Gießen, Germany; 2 Department of Anaesthesiology and Intensive Care Medicine, University Hospital of Gießen, Germany; 3 Institute of Pathology, University Hospital of Gießen, Germany; 4 Department of General and Visceral Surgery, Düren Hospital, Düren, Germany

## Abstract

**Background:**

Gastrointestinal stromal tumors (GISTs) are the most common mesenchymal tumors of the digestive tract. The majority of GISTs is located in the stomach. Only 3-5% of GISTs are located in the duodenum associated with an increased risk of gastrointestinal bleeding as primary manifestation. With response rates of up to 90%, but complications like bleeding due to tumor necrosis in 3%, imatinib mesylate dramatically altered the pre- and postoperative therapy for GIST patients.

**Case presentation:**

A 58-year-old female patient presented with acute upper gastrointestinal bleeding 2 weeks after a giant GIST of the duodenum had been diagnosed. Neoadjuvant imatinib therapy had been initiated to achieve a tumor downsizing prior to surgery. During emergency laparotomy a partial duodenopancreatectomy was performed to achieve a complete resection of the mass. Histology revealed a high-malignancy GIST infiltrating the duodenal wall. Adjuvant imatinib therapy was initiated. At follow-up (19 months) the patient is still alive and healthy.

**Conclusion:**

Giant GISTs of the duodenum are rare and - in contrast to other localizations - harbour a higher risk of serious bleeding as primary manifestation. Tumor necrosis and tumor bleeding are rare but typical adverse effects of imatinib therapy especially during treatment of high-malignancy GIST. In GIST patients with increased risk of tumor bleeding neoadjuvant imatinib therapy should thoroughly be performed during hospitalization. In cases of duodenal GIST primary surgery should be considered as treatment alternative.

## Background

Gastrointestinal stromal tumors (GISTs) are the most common mesenchymal tumors of the gastrointestinal wall. Originating in the muscular wall of the viscera proliferating cells of a GIST show phenotypic similarities to the interstitial cells of Cajal, which coordinate the peristaltic movements of the gastrointestinal tract [1,2]. GISTs are defined as mesenchymal, spindle-shaped tumors, which can be distinguished from other soft tissue tumors like leiomyomas, myoblastomas etc. by c-kit protooncogen (CD117) expression [3]. With 33-63% the stomach is the most common site for a GIST, followed by the small intestine (23-38%) and the colorectal localization (5-32%). In contrast GISTs of the duodenum, as presented in this case report, are very rare.

Clinical studies demonstrated that with imatinib (STI 571, Gleevec, Novartis Pharma) objective responses can be reached in more then 50% of patients with an advanced GIST. Unresectable locally advanced tumors, recurrent or metastatic GISTs showed longer progression-free survival under imatinib therapy [4-6]. All in all 80-90% of the patients with GISTs showed at least a partial tumor response [7]. After retrospective small-institutional reports and case series the neoadjuvant use of imatinib in GIST was first evaluated in the RTOG 0132/ACRIN 6665 study. Eisenberg recently published first results underlining the safety of imatinibmesylate in treatment of GISTs [8]. In another study 3% of the probands treated with imatinib developed complications caused by rupture of large tumor masses which became necrotic unter pharmacotherapy [9]. These data correspond to complication rates described in the STI 571 study [9,10]. To the best of our knowledge these complication rates only referred to common tumor localizations, but not to uncommon GIST sites as duodenum, rectum or others [8].

We herein report a case of a patients with a giant GIST of the duodenum. After neoadjuvant imatinib therapy was initiated, a dramatic tumor regression led to an upper gastrointestinal bleeding and an emergency laparotomy.

## Case Report

The 58-year-old female patient was hospitalized due to recurrent episodes of upper abdominal pain, anemia, weight loss, fatigue and fever attacks. Under suspicion of a duodenal perforation by a lymphoma or GIST, seen in an ultrasound examination, the patient was transferred to our clinic.

Physical examination of the patient with no history of preexisting diseases revealed a palpable mass in the right upper abdominal quadrant. Hemoglobin was 90 g/l.

**Figure 1:**
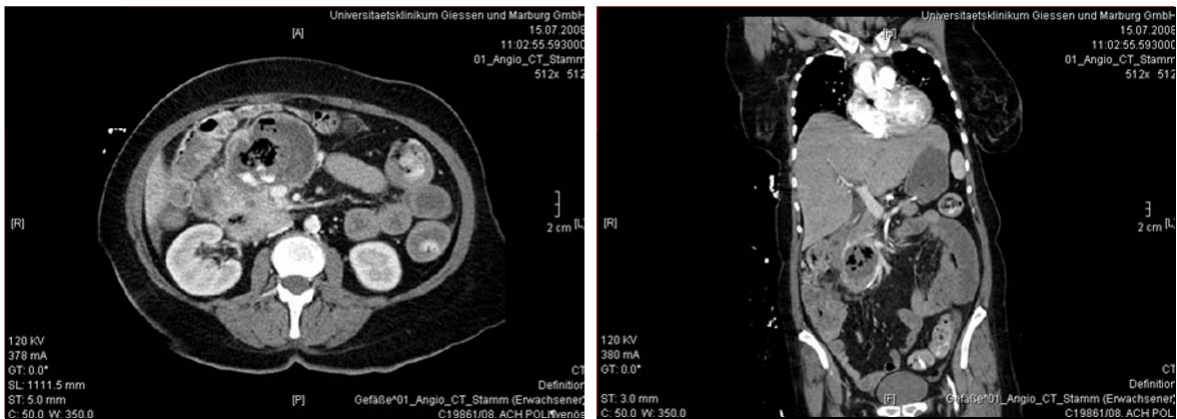
**Transversal (left) and coronal (right) CT scans of the abdomen reveal a cystic and necrotic tumor cavity 2 weeks after initiation of imatinib therapy**.

**Figure 2:**
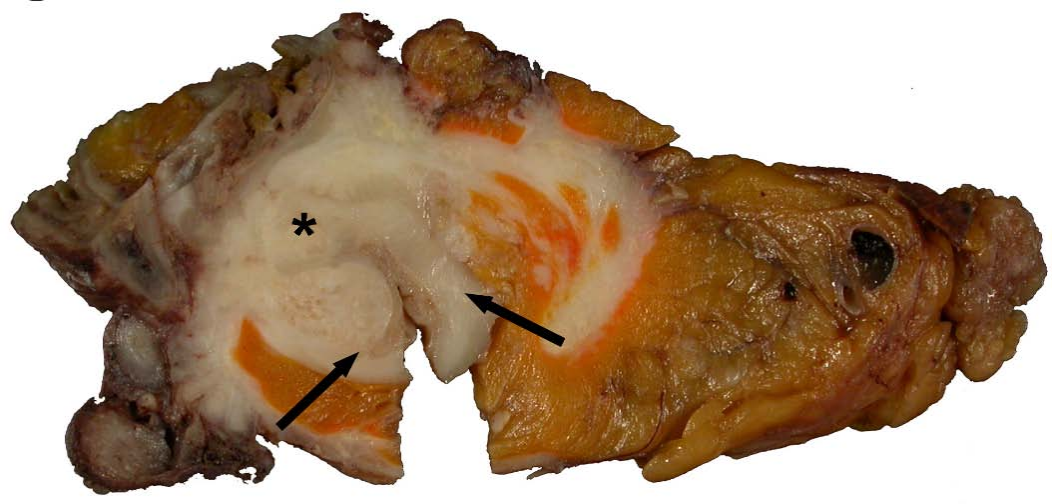
**Cross-section of the surgical specimen showing the tumor (asterisk) infiltrating the duodenal wall (arrows)**.

Upper endoscopy revealed a large necrotic cavity in the inferior part of the duodenum. Multiple biopsies taken from the tumor mass confirmed the suspicion of a duodenal GIST. PET-CT scan showed a 9 × 9 × 15 cm tumor mass arising from the duodenum with a maximal standard uptake value (SUV) of 15,5. The tumor had contact to the pancreatic caput and led to compression of the inferior caval vein and the inferior mesenterial vein. The portal vein as well as the common hepatic artery and the superior mesenterial artery showed no signs of infiltration or compression. Furthermore PET-CT did not reveal any signs of metastasis. According to a neoadjuvant approach preoperative therapy with imatinib (Gleevec, Novartis, Basel, Switzerland), 400 mg per day, was initiated immediately. Responder controll by PET-CT scan was planned to be performed 4 weeks after initiation of the therapy. After 2 weeks under ambulatory pharmacological therapy the patient presented in the emergency room with an acute upper gastrointestinal bleeding. CT confirmed a dramatic bleeding from the upper GI tract necessitating mass blood transfusion (Fig. 1). Tumor size decreased to 7 × 8 × 12 cm within only 2 weeks of imatinib treatment. An angiographic CT showed the diffuse tumor bleeding supplied by the gastroduodenal artery and some branches of the superior mesenterial artery. The diffuse bleeding forbade a coiling of the vessels. During the emergency laparotomy an encapsulated tumor mass could be identified, originating from the descendent part of the duodenum and reaching both the pancreatic caput and the right flexure of the colon. Obviously the giant tumor had led to a bleeding by arrosion of peripancreatic vessels. After ligation of the vessels supplying the mass a partial pancreaticoduodenectomy (Traverso-Longmire) was performed to resect the tumor (Fig. 2). Additionally a resection of the right hemicolon was performed due to tumor infiltration of the right curvature of the colon. Continuity was reconstructed by gastrojejunostomy (Traverso-Longmire) on the one hand and an end-to-side-pancreaticojejunostomy on the other hand. An ileotransversostomy was performed to reconstruct the gastrointestinal passage.

**Figure 3:**
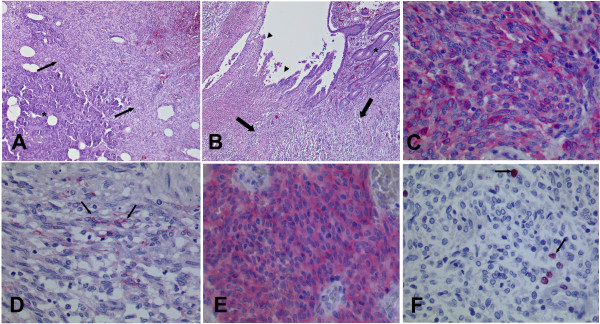
**A) GIST infiltrating adjacent Pancreas; asterisk = pancreatic glands and ducts, arrows = GIST [hematoxylin-eosin staining, Original magnification 50×]**. B) GIST perforating into the lumen of the duodenum; asterisk = mucosa of the duodenum, arrowheads = ulceration, arrows = GIST [hematoxylin-eosin staining, Original magnification 50×] C) Immunohistochemical staining for Vimentin showing positive reaction in almost all cells (red colour) [Original magnification 400×] D) Cells of the GIST with spindle-shaped nuclei (arrows) and admixed lymphocytes and granulocytes [hematoxylin-eosin staining, Original magnification 400×] E) Immunohistochemical staining for CD117 (c-kit) showing positive reaction in almost all cells (red colour) [Original magnification 400×] F) Immunohistochemical staining for Ki-67 showing proliferation in approximately 5-10% of cells (red nuclear signal, arrows) [Original magnification 400×].

Upon macroscopic examination the specimen showed a partially necrotic mesenchymal mass with a diameter of 9 cm, an infiltration of the duodenal wall leading to ulceration and perforation, an infiltration of the pancreas and two peripankreatic tumor islands (Fig. 2). There were no signs of metastases in locoregional lymphnodes. Histological examination of the tumour tissue revealed the typical appearance of a GIST composed of cells with spindle-shaped nuclei (Fig.3D). Immunohistochemically the tumour cells showed an expression of Vimentin (Fig. 3C) and CD117 (Fig. 3E), a focal expression of CD34, smooth-muscle-actin (not shown) and a nuclear expression of the proliferation-associated Ki-67-antigen in approximately 5-10% of the tumour cells (Fig. 3F). The tumour was negative for S-100 and Keratin (not shown).

Two days after surgery the patient was weaned and successfully extubated. After an uneventfull recovery the patient is alive and without any signs of tumor recurrence. Up to the follow-up of 19 months the patient permanently received an adjuvant imtinib therapy (400 mg per day).

## Discussion

GISTs are defined as mesenchymal tumors arising from the gastrointestinal wall, mesentery, omentum or retroperitoneum. In contrast to leiomyo(sarko)mas GIST cells express the c-kit proto-oncogene (CD117). Distribution of GIST in the gastrointestinal tract was analyzed in several studies. Tumors are mostly localized in the stomach (33-63%), small bowel (23-38%), whereas colon, rectum and esophagus are rare localizations. The female patient of this case report presented with a duodenal GIST as another rare GIST manifestation. Except for one large study on the histopathological pattern of duodenal GIST [11] only two studies with 8 and 15 patients respectively are published so far [12,13] analyzing the clinic and the outcome of duodenal GIST patients. Compared to other tumor localizations duodenal GISTs manifestate with tumor-associated bleeding in 90 resp. 75% compared to 54% (stomach) [14] and 28% (ileo-jejunal) [15]. In contrast to other localizations duodenal GISTs are thus associated with a dramatically increased risk of an upper intestinal bleeding [11].

Nowadays the dignity of resected tumors is classified in risk categories that are based on size and mitotic rate mainly: In a consensus approach Fletcher et al. came to the result that tumor size (>5 cm) and mitotic activity (>5/50 high-power field) of the mesenchymal cells are the most important independent prognostic factors for tumor progression [3]. In our case postoperative examination of the specimen revealed a tumor mass of 9 × 15 cm in diameter. Ki67 was used as an immunhistochemical marker for cell proliferation. 5-10% of the tumor cells were Ki67^+^. Histopathological examination revealed a rate of 12 mitoses per 50 high power fields. Following the above-mentioned classification our patient fulfilled all criteria of a malignant tumor progress.

Surgical therapy of duodenal GIST depends on tumor localization and is either partial duodenectomy, or partial pancreaticoduodenectomy. Interestingly, a recent study revealed that duodenal GIST cells express a different pattern of immunhistochemical markers [16]. Additionally authors showed that duodenal GIST are associated with a more favorable prognosis compared to other tumor localizations. [16]. After review of recent literature the duodenal tumor localization in our case is thus associated with a better prognosis, but with an increased bleeding probability. These results are in line with the authors' opinion that primary surgery could be the safest therapeutic option for a GIST of this localization. Beside the increased risk of tumor bleeding, caused by the localization, neoadjuvant imatinib therapy would additionally lead to a higher percentage of patients with a tumor bleeding.

Without any pre- or postoperative pharmacotherapy complete surgical resection can lead to a 5 year survival of up to 45% [17]. The introduction of imatinib mesylate, a tyrosine kinase inhibitor targeting KIT has provided a much needed chemotherapeutical option for patients with both resectable and irresectable GISTs. Despite the noted success of imatinib surgical resection is the main treatment modality for primary GIST of any localization. Imatinib treatment of GISTs is a dynamic process with the permanent risk of pharmacoresistance [4,18] and a maximal respondance within the first 6 months of therapy [4]. Nevertheless the benefit of adjuvant imatinib for primary GIST has been underlined by several trials completed recently. As a consequence surgeons started to use imatinib in a neoadjuvant therapy to reach tumor-downsizing and thus increased rates of complete tumor resection.

As mentioned above complications like tumor necrosis and tumor bleeding occurr in 3% of patients under imatinib therapy. These data are in line with results of the STI 571 study. In the prospective RTOG study Eisenberg reports on a minimal rate of toxicity or of intraooperative complications under neoadjuvant imatinib therapy in 30 cases [8]. Nevertheless only 1 of 30 patients with imatinib therapy prior to surgery had a duodenal GIST [8]. It thus remains unclear, if the high safety of imatinib can be transferred to patients with GISTs at rare tumor localizations. Alternatively primary surgery should be seen as an alternative therapeutic approach. In cases of duodenal GIST primary surgery could be supported by a preoperative transarterial embolization as published previously [19].

## Conclusions

(1) The review of literature reveals a higher rate of tumor bleeding as primary tumor manifestation in cases of duodenal GIST. (2) Beside the rare and uncommon localization we describe the case of a giant GIST of highest malignancy according to the classification of Fletcher et al.(3). Imatinib led to revolutionary changes in therapy of primary and metastatic GIST, but is associated with rapid tumor regression, necrosis and tumor bleeding in 3%. For the case presented a higher complication rate is to be exspected.

In GIST patients with these localizations and thus an increased risk of tumor bleeding neoadjuvant imatinib therapy should thoroughly be performed during hospitalization. In cases of duodenal GIST primary surgery should be considered as treatment alternative.

## Competing interests

The authors declare that they have no competing interests.

## Authors' contributions

AH and BH equally contributed to this study and were responsible for data collection and analysis of the patient's case. Furthermore they wrote the manuscript. BB performed the histopathological examination of the resected specimen. TS is the corresponding author of this study. He is responsible for the paper format and submission. MH and HJ were surgeons treating the patient. They helped to draft the manuscript. WP is the director of the department. He designed the study. All authors read and approved the final manuscript.

## Consent

Written informed consent was obtained from the patient for publication of this case report and accompanying images. A copy of the written consent is available for review by the Editor-in-Chief of this journal.

## References

[B1] Kindblom LG Remotti HE Aldenborg F Meis-Kindblom JM Gastrointestinal pacemaker cell tumor (GIPACT): gastrointestinal stromal tumors show phenotypic characteristics of the interstitial cells of Cajal Am J Pathol 1998 152 1259 1269 9588894 PMC1858579

[B2] Miettinen M Sarlomo-Rikala M Lasota J Gastrointestinal stromal tumors: recent advances in understanding of their biology Hum Pathol 1999 30 1213 1220 10.1016/S0046-8177(99)90040-0 10534170

[B3] Fletcher CD Berman JJ Corless C Gorstein F Lasota J Longley BJ Miettinen M O'Leary TJ Remotti H Rubin BP Shmookler B Sobin LH Weiss SW Diagnosis of gastrointestinal stromal tumors: A consensus approach Hum Pathol 2002 33 459 465 10.1053/hupa.2002.123545 12094370

[B4] Verweij J Casali PG Zalcberg J LeCesne A Reichardt P Blay JY Issels R van Oosterom A Hogendoorn PC Van Glabbeke M Bertulli R Judson I Progression-free survival in gastrointestinal stromal tumours with high-dose imatinib: randomised trial Lancet 2004 364 1127 1134 10.1016/S0140-6736(04)17098-0 15451219

[B5] van Oosterom AT Judson I Verweij J Stroobants S Donato di Paola E Dimitrijevic S Martens M Webb A Sciot R Van Glabbeke M Silberman S Nielsen OS Safety and efficacy of imatinib (STI571) in metastatic gastrointestinal stromal tumours: a phase I study Lancet 2001 358 1421 1423 10.1016/S0140-6736(01)06535-7 11705489

[B6] Demetri GD von Mehren M Blanke CD Van den Abbeele AD Eisenberg B Roberts PJ Heinrich MC Tuveson DA Singer S Janicek M Fletcher JA Silverman SG Silberman SL Capdeville R Kiese B Peng B Dimitrijevic S Druker BJ Corless C Fletcher CD Joensuu H Efficacy and safety of imatinib mesylate in advanced gastrointestinal stromal tumors N Engl J Med 2002 347 472 480 10.1056/NEJMoa020461 12181401

[B7] Katz SC DeMatteo RP Gastrointestinal stromal tumors and leiomyosarcomas J Surg Oncol 2008 97 350 359 10.1002/jso.20970 18286477

[B8] Eisenberg BL Harris J Blanke CD Demetri GD Heinrich MC Watson JC Hoffman JP Okuno S Kane JM von Mehren M Phase II trial of neoadjuvant/adjuvant imatinib mesylate (IM) for advanced primary and metastatic/recurrent operable gastrointestinal stromal tumor (GIST): Early results of RTOG 0132/ACRIN 6665 J Surg Oncol 2009 99 42 47 10.1002/jso.21160 18942073 PMC2606912

[B9] Bechtold RE Chen MY Stanton CA Savage PD Levine EA Cystic changes in hepatic and peritoneal metastases from gastrointestinal stromal tumors treated with Gleevec Abdom Imaging 2003 28 808 814 10.1007/s00261-003-0021-2 14753595

[B10] van Oosterom AT Judson IR Verweij J Stroobants S Dumez H Donato di Paola E Sciot R Van Glabbeke M Dimitrijevic S Nielsen OS Update of phase I study of imatinib (STI571) in advanced soft tissue sarcomas and gastrointestinal stromal tumors: a report of the EORTC Soft Tissue and Bone Sarcoma Group Eur J Cancer 2002 38 Suppl 5 S83 87 10.1016/S0959-8049(02)80608-6 12528778

[B11] Miettinen M Kopczynski J Makhlouf HR Sarlomo-Rikala M Gyorffy H Burke A Sobin LH Lasota J Gastrointestinal stromal tumors, intramural leiomyomas, and leiomyosarcomas in the duodenum: a clinicopathologic, immunohistochemical, and molecular genetic study of 167 cases Am J Surg Pathol 2003 27 625 641 10.1097/00000478-200305000-00006 12717247

[B12] Winfield RD Hochwald SN Vogel SB Hemming AW Liu C Cance WG Grobmyer SR Presentation and management of gastrointestinal stromal tumors of the duodenum Am Surg 2006 72 719 722 discussion 722-713 16913316

[B13] Goh BK Chow PK Kesavan S Yap WM Wong WK Outcome after surgical treatment of suspected gastrointestinal stromal tumors involving the duodenum: is limited resection appropriate? J Surg Oncol 2008 97 388 391 10.1002/jso.20954 18163461

[B14] Miettinen M Sobin LH Lasota J Gastrointestinal stromal tumors of the stomach: a clinicopathologic, immunohistochemical, and molecular genetic study of 1765 cases with long-term follow-up Am J Surg Pathol 2005 29 52 68 10.1097/01.pas.0000146010.92933.de 15613856

[B15] Miettinen M Makhlouf H Sobin LH Lasota J Gastrointestinal stromal tumors of the jejunum and ileum: a clinicopathologic, immunohistochemical, and molecular genetic study of 906 cases before imatinib with long-term follow-up Am J Surg Pathol 2006 30 477 489 10.1097/00000478-200604000-00008 16625094

[B16] Yang WL Yu JR Wu YJ Zhu KK Ding W Gao Y Shen QY Lv KZ Zhang Q Yang XJ Duodenal gastrointestinal stromal tumor: Clinical, pathologic, immunohistochemical characteristics, and surgical prognosis J Surg Oncol 2009 19697360 10.1002/jso.21378

[B17] DeMatteo RP Lewis JJ Leung D Mudan SS Woodruff JM Brennan MF Two hundred gastrointestinal stromal tumors: recurrence patterns and prognostic factors for survival Ann Surg 2000 231 51 58 10.1097/00000658-200001000-00008 10636102 PMC1420965

[B18] Van Glabbeke M Verweij J Casali PG Le Cesne A Hohenberger P Ray-Coquard I Schlemmer M van Oosterom AT Goldstein D Sciot R Hogendoorn PC Brown M Bertulli R Judson IR Initial and late resistance to imatinib in advanced gastrointestinal stromal tumors are predicted by different prognostic factors: a European Organisation for Research and Treatment of Cancer-Italian Sarcoma Group-Australasian Gastrointestinal Trials Group study J Clin Oncol 2005 23 5795 5804 10.1200/JCO.2005.11.601 16110036

[B19] Kurihara N Kikuchi K Tanabe M Kumamoto Y Tsuyuki A Fujishiro Y Otani Y Kubota T Kumai K Kitajima M Partial resection of the second portion of the duodenum for gastrointestinal stromal tumor after effective transarterial embolization Int J Clin Oncol 2005 10 433 437 10.1007/s10147-005-0503-z 16369749

